# Providing trial results to participants in phase III pragmatic effectiveness RCTs: a scoping review

**DOI:** 10.1186/s13063-021-05300-x

**Published:** 2021-05-24

**Authors:** Hanne Bruhn, Elle-Jay Cowan, Marion K. Campbell, Lynda Constable, Seonaidh Cotton, Vikki Entwistle, Rosemary Humphreys, Karen Innes, Sandra Jayacodi, Peter Knapp, Annabelle South, Katie Gillies

**Affiliations:** 1 Health Services Research Unit, Health Sciences Building, Foresterhill, Aberdeen, UK; 2 Public Partner, Devon, UK; 3 Public Partner, London, UK; 4 Department of Health Sciences, Seebohm Rowntree Building, University of York and the Hull York Medical School, York, UK; 5 MRC Clinical Trials Unit at UCL, 90 High Holborn, London, UK

**Keywords:** Clinical trials, Results, Dissemination, Participants

## Abstract

**Background:**

There is an ethical imperative to offer the results of trials to those who participated. Existing research highlights that less than a third of trials do so, despite the desire of participants to receive the results of the trials they participated in. This scoping review aimed to identify, collate, and describe the available evidence relating to any aspect of disseminating trial results to participants.

**Methods:**

A scoping review was conducted employing a search of key databases (MEDLINE, EMBASE, PsycINFO, and the Cumulative Index to Nursing & Allied Health Literature (CINAHL) from January 2008 to August 2019) to identify studies that had explored any aspect of disseminating results to trial participants. The search strategy was based on that of a linked existing review. The evidence identified describes the characteristics of included studies using narrative description informed by analysis of relevant data using descriptive statistics.

**Results:**

Thirty-three eligible studies, including 12,700 participants (which included patients, health care professionals, trial teams), were identified and included. Reporting of participant characteristics (age, gender, ethnicity) across the studies was poor. The majority of studies investigated dissemination of aggregate trial results. The most frequently reported mode of disseminating of results was postal. Overall, the results report that participants evaluated receipt of trial results positively, with reported benefits including improved communication, demonstration of appreciation, improved retention, and engagement in future research. However, there were also some concerns about how well the dissemination was resourced and done, worries about emotional effects on participants especially when reporting unfavourable results, and frustration about the delay between the end of the trial and receipt of results.

**Conclusions:**

This scoping review has highlighted that few high-quality evaluative studies have been conducted that can provide evidence on the best ways to deliver results to trial participants. There have been relatively few qualitative studies that explore perspectives from diverse populations, and those that have been conducted are limited to a handful of clinical areas. The learning from these studies can be used as a platform for further research and to consider some core guiding principles of the opportunities and challenges when disseminating trial results to those who participated.

**Supplementary Information:**

The online version contains supplementary material available at 10.1186/s13063-021-05300-x.

## Background

Reporting of trial results to those who participated is a fundamental requirement of ethical trials, underscored in the 2013 version of the Declaration of Helsinki which states ‘Researchers, authors, sponsors, editors and publishers all have ethical obligations with regard to the publication and dissemination of the results of research.’ and ‘All medical research subjects should be given the option of being informed about the general outcome and results of the study.’ [1]. In a recent survey of authors of clinical trials, 27% reported having disseminated results to participants, 13% planned to do so, but 33% had no intention of communicating the results to their participants (with a further 10% stating they were unsure, and the final 17% indicating ‘other’ or not answering) [2]. Other research indicates that, of those who do intend to share results, often this is operationalised passively, puts the onus on participants to request or access information and provides information in forms that may be difficult to access or understand (e.g. scientific publication) [3]. Clinical trial registers, such as ClinicalTrials.gov, indicate that there are hundreds of thousands of trials currently registered (https://clinicaltrials.gov/ct2/resources/trends). If only 27% of these registered trials are returning results to participants, this is a significant problem. Most research participants want to be informed about the results of the study they participated in [4]. These figures suggest poor practice that requires urgent attention.

It is clear that the research community need to know how to do this better. A range of barriers have been reported to impact on trial teams’ abilities to disseminate results to participants [2]. These include concerns that patients do not want or will not understand results, uncertainty about what results to share and with whom and when, difficulty reaching patients, lack of early planning and support, lack of academic expectation or incentive and concerns related to researchers lack of experience [2]. Several changes have been proposed in order to improve rates of sharing of trial findings with participants and/or improve how the findings are shared but a multi-factorial multi-stakeholder approach will be required to make this common practice [2]. One of the key considerations to facilitate dissemination is the provision of evidence based practical guidance on when, what and how to share results with trial participants for which there have been several calls [2, 5].

A previous review of how results have been disseminated to research participants has been published, but is now several years out of date [4]. Furthermore, the review considered research more generally and did not focus on particular study designs and how these might influence the what, when and how of dissemination [4]. It is likely that different research designs may vary in the challenges and opportunities for providing results to those who participated for example, in some trials, people do not know what treatment they received, which raises questions about whether to reveal that information or not. Clinical trials, and in particular phase III pragmatic effectiveness randomised controlled trials (RCTs) are an important place to start because they usually enrol the largest number of trial participants (due to them being confirmatory trials), and therefore, any recommendations developed will have the widest potential reach. Detailed understanding of one major study design used in a diverse research context will provide a baseline platform on which to generate good practice that can be applied to other settings. In addition, given the current spotlight on research transparency, it is critical that we provide up to date evidence summaries that can inform the development of dissemination of trial results to participants [6].

As a first step to generate evidence-based recommendations for triallists to implement the dissemination of results to trial participants, there is a need to collate the evidence on what research has been done to date. The purpose of the evidence review outlined in this manuscript was to identify the breadth of research, not determine which method is ‘best’; thus, a scoping review was conducted. Scoping reviews aim to ‘systematically map the literature available on a topic, identifying key concepts, theories, sources of evidence and gaps in the research’ [7]. They are useful to explore breadth and depth across heterogeneous literature [7, 8].

This scoping review aimed to identify, collate and describe the available evidence relating to any aspect of disseminating trial results to participants both in terms of study characteristics and key features of the dissemination activity (namely what information to communicate, how to communicate it and reported advantages/disadvantages). The review had two objectives:

1. To develop an overview that identifies and characterises published research studies that have investigated any aspect of disseminating results to participants of phase III RCTs.
2. To identify evidence gaps where replication of evaluations or initiation of new research could be of value and to provide recommendations to directly inform research linked to this review as part of a programme of funded work (RECAP: Reporting Clinical trial results Appropriately to Participants [9]) to develop evidence-based recommendations that are attentive to diverse participants’ experiences and preferences.

## Methods

The work reported in this review relates to phase 1 of RECAP and specifically the identification of methods used to disseminate results to trial participants and reporting the key features of such methods. This scoping review was conducted and reported in accordance with the relevant items for scoping reviews specified in the Preferred Reporting Items for Systematic Reviews and Meta-analysis extension for Scoping Reviews (PRISMA - ScR) checklist (See Supplementary Table 1).

### Search strategy

The search strategy was developed in consultation with a Senior Information Scientist and KG and informed by from a previous review in this area [4]. A systematic search of the literature was conducted across MEDLINE, EMBASE, PsycINFO, and the Cumulative Index to Nursing & Allied Health Literature (CINAHL) from January 2008 to August 2019. Dates for the search commenced from 2008 as a previous review by Shalowitz and Miller had been conducted and captured relevant published studies up to 2008. The review by Shalowitz and Miller aimed to report on the trends in practice with regard to sharing results in terms of content, and stakeholder attitudes and so was deemed similar enough in scope to warrant it being used as a platform to identify studies published pre-2008 [4]. Studies identified in this previously published review that were relevant to phase III trials were identified from the reference list and included for data extraction. A full-search strategy is available in Appendix. Conference abstracts were included in the search and citation searching of systematic reviews identified was also conducted. We also searched the Studies Within Trials (SWAT) repository for ongoing studies. We did not contact relevant authors.

### Inclusion and exclusion criteria

Reports eligible for inclusion included protocols, systematic reviews with/without meta-analysis, reports of RCTs, quantitative or qualitative studies and reports describing the process of results provision (both aggregate and individual) to trial participants. Reports known to the authors but not identified by the search (*n*=10, including some of the papers identified in the previous pre-2008 review which were relevant for trials [4]) were also included in the pool of potentially eligible studies and assessed using the inclusion and exclusion criteria. Included studies had to report data from, or information about, trials that recruited adult participants (aged 16 years and older), but these participants could be any trial stakeholder (e.g. REC members, trialists, funders, sponsors, representatives from industry, members of the public and/or current or previous participants in trials). Studies had to meet minimum eligibility criteria for inclusion. These criteria were that the study had to be about provision of results within the context of phase III pragmatic effectiveness trials in non-emergency settings. If the phase of trial was not clear the studies were still included, however, if studies included multiple phases of trial and it was not clear which data related to which phase the study was excluded. The decision to exclude results in emergency settings related to the role of proxies in the consent process and potentially the same individuals receiving results for trials in this setting. We believed this setting may influence what information is/has been shared and decided to focus on trials in non-emergency settings as a starting point. Any interventions relating to dissemination of results were considered eligible as were any outcome assessments of effectiveness or indeed qualitative findings relevant to results dissemination. Studies were excluded based on the following criteria: papers reporting on provision of results in non-trial research; reports relating to phase I, II or IV trials specifically; reports using hypothetical trial scenarios; and non-empirical articles (e.g. commentaries).

### Eligibility of studies

Titles and abstracts identified in the search were independently assessed by one reviewer (HB) with other reviewers (AS, SC, KG) double screening the search output. Any disagreement between abstract screeners on the eligibility of included papers was resolved through discussion. Full-text papers were obtained where applicable for those studies that on initial screening were considered potentially relevant and were further assessed for inclusion by one reviewer (HB) with queries resolved through discussion (KG). Posters were sought for relevant conference abstracts, etc. Papers pre-2008 were identified from reference [4] and the eligibility criteria applied and assessed as above.

### Data extraction

A data extraction form was developed by the study team in advance of data collection. It captured information on study characteristics (e.g. population, setting, study design, participant characteristics) and data directly relevant to provision of trial results: description of the intervention disseminating results in relevant studies, description of results provided (i.e. aggregate vs individual results), mode, content, how content was developed/decided, description of PPI involvement, timing (e.g. before or after publication of results), who delivered trial results, any outcomes reported (i.e. of the evaluation of the results intervention), and reported advantages and disadvantages of results provision. The data extraction form was piloted on three papers. Data from all included studies were extracted by one reviewer (EC) with a random 40% of studies double data extracted (HB, KI, SC and LC) and checked for consistency (KG). Authors of included studies were not contacted for further information or verification.

### Data analysis

Data from the included studies were analysed using descriptive statistics with overall findings presented using narrative summary. As this was a scoping review, no formal critical appraisal of the quality of included studies was conducted, which is in line with published guidance for scoping reviews [7, 8].

## Results

### Search results

**Fig. 1:**
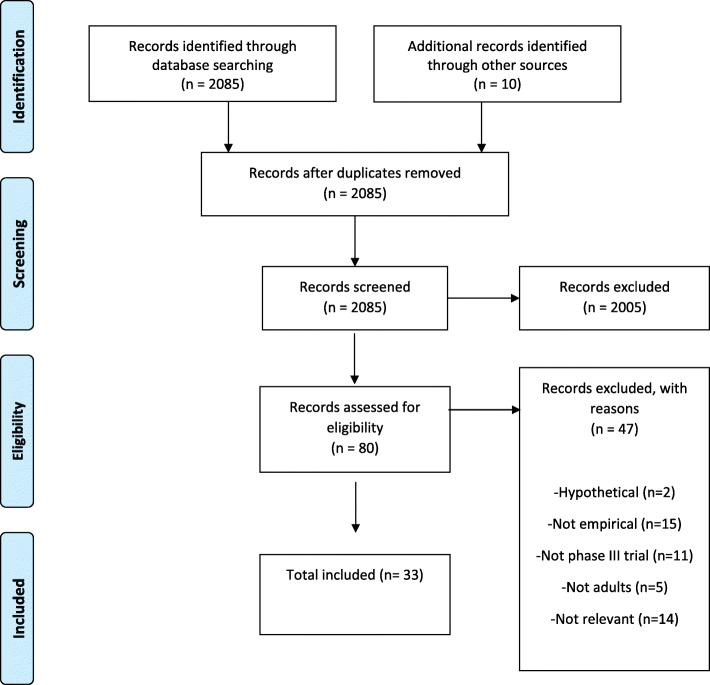
PRISMA flow diagram

After removing duplicates, the searches run from January 2008 to August 2019 identified a total of 2085 studies as potentially eligible and included for further assessment and inclusion in the review. The majority of these reports (*n*=2005) were excluded due to not meeting the minimum eligibility criteria, providing 80 papers for a full-text review. Of these, a further 47 were excluded due to using hypothetical trial scenarios (*n*=2), not recruiting adults (*n*=5) not being phase III trials (*n*=11), not being empirical studies (*n*=15), or not being about results dissemination (*n*=14) (Fig. 1). The remaining 33 reports were deemed eligible for inclusion in the review and progressed to data extraction. The 33 included papers reported studies from 27 trials, with the majority of studies (*n*=24) linked to separate trials [10–42]. See further detials of characteristics of included studies in Table 1.

**Table 1:** Included studies characteristics

Study (first author and year of publication)	Study title	Population (trial participants/generic/staff)	Number of study participants	Sex (male/female)	Age	Setting (secondary/ primary care/community)	Country	Aim (verbatim from included papers)	Design	Type (aggregate /individual both)	Timing (when results were provided)
Garcia 1987 [10]	Sharing research results with patients: the views of care-givers involved in a randomised controlled trial	HCP	16	Not specified	Not specified	Secondary care	Ireland	To explore views of doctors and midwives in a large maternity hospital who were involved in a randomised controlled trial (to compare methods of monitoring the foetal heart during labour) were asked whether the trial results should be passed on to women coming to the hospital for their care.	Interviews	N/A	N/A
Di Blasi 2002 [11]	Informing participants of allocation to placebo at trial closure: postal survey	Investigators	212	Not specified	Not specified	N/A	UK	To assess whether and how investigators of placebo controlled randomised trials inform participants of their treatment allocation at trial closure and to assess barriers to feedback.	Postal survey with a semi-structured questionnaire	Individual	N/A
Partridge 2004 [12]	Oncology physician and nurse practices and attitudes regarding offering clinical trial results to study participants	HCP	796	Not specified	Not specified	N/A	USA	To determine the practices attitudes of oncology physician and nurses regarding offering clinical trial results to study participants.	Mailed survey	N/A	N/A
Dinnett 2005 [13]	Unblinding of trial participants to their treatment allocation: lessons from the prospective study of pravastatin in the elderly at risk (PROSPER)	Trial participants	1492	Not specified	Not specified	Secondary care	UK	The aim of the study was to determine whether the unblinding process could be conducted: (1) in an efficient manner, all study participants with their study medication status and on-trial cholesterol levels; (2) to sensitively and with the support of counselling where appropriate; (3) to respect the rights of participants not to be unblinded.	Postal survey	Individual	Simultaneously with publication
Dixon-Woods 2006 [14]	Receiving a summary of the results of a trial: qualitative study of participant’ views	Trial participants	20	Female	Not specified	Secondary care	UK	To explore trial participants’ responses to receiving a summary of the results of a trial in pregnancy.	Qualitative study with semi-structured interviews.	Aggregate	Simultaneously with publication
Avins 2008 [15]	Initial experience with a group presentation of study results to research participants	Trial participants	225	Male	>50	Secondary care	USA	To document experience with a group presentation of the results of a clinical trial and ways in which this process could be adapted and improved in future studies.	A survey was conducted at the second presentation that assessed the value and perceptions of the meeting, addressing knowledge learned about the study from the presentation and the published paper; no formal validation of the survey instrument was conducted.	Aggregate	After publication
Dorsey 2008 [16]	Communicating clinical trial results to research participants	Trial participants	114	Both	Mean age 55.1 years	Not specified	USA and Canada	To evaluate the effectiveness of a plan to communicate results in an industry-sponsored randomised controlled trial for Huntington disease.	Postal survey	N/A	Simultaneously with press release
Johnson 2008 [17]	How do patients want to learn of results of clinical trials? A survey of 1431 breast cancer patients	Trial participants and HCP	1431	Female	% 18-29, 76% 40–59, 15% 60–69; 1% 70+	Secondary care	UK and Belgium	To find out from trial patients whether they wanted to receive trial results written in lay terms when they are available, and how they considered they wanted to receive them. We compare their preferences with those expressed by health care professionals (oncologists and nurses) who had participated in the TACT trial.	Questionnaire	Aggregate	Not specified
Darbyshire 2009 [18]	Presenting the results of clinical trials to participants	Trial participants	140	Not specified	Not specified	Secondary care	UK and Ireland	To generate evidence from it a review how the 1-year results from the 4-T Trial were published and subsequently discussed with participants to inform further work.	Information was retrospectively collected from clinical centres who held a coffee morning to disseminate the one-year results from the three year. Treat to Target in Type 2 Diabetes (4-T) Trial (Current Controlled Trials number, ISRCTN51125379). Following a discussion of the results, suggestions for how the information collected could be used to inform further work in this area were made.	Aggregate	Simultaneously with publication
Partridge 2009 [19]	The impact of sharing results of a randomised breast cancer clinical trial with study participants	Trial participants	167	Female	Mean age 51 years	Secondary care	USA	We sought to evaluate patient perceptions of how results had been shared from a large randomised cooperative group trial, and the impact of learning results.	Mailed surveys	Aggregate	Not specified
Brealey 2010 [20]	Participants’ preference for type of leaflet used to feed back the results of a randomised trial: a survey	Trial participants	132	Both	Mean age 43 years	Primary care	UK	This study aims to determine participants’ preferences for type of leaflet (short versus long) used to summarise the findings of a randomised trial; and to test whether certain characteristics explained participants’ preferences	Questionnaire	Aggregate	After publication
Dalal 2010 [21]	Communicating the results of research: how do participants of a cardiac rehabilitation RCT prefer to be informed	Trial participants	154	Both	Mean age 68.5 years	Primary care	UK	To determine the preferred means by which participants in a study of cardiac rehabilitation wish to be informed of the study’s results.	Postal questionnaire survey of participants in a RCT.	Aggregate	After publication
Getz 2010 [22]	Celebrex (NCT00139776) and Sutent (NCT00137449)	Trial participants	13	Both	Not specified	Secondary care	USA	To evaluate the feasibility of integrating a post-trial communication mechanism with ongoing clinical development activities; to develop and test the comprehension and impact of various communication formats and messages; and to assess perceptions and reactions to this new post-trial communication process among clinical research professionals	Pilot study-study volunteers in focus groups	Aggregate	After publication
Mancini 2010 [23]	FNCLCC-PACS04	Trial participants	115	Female	Mean age 56.7 years	Not specified	France	The aim of this study was to assess patients’ uptake and understanding of the results of the trial in which they have participated, and the impact of a letter offering patients the possibility of consulting the trial results on a specific website	Randomised trial Ppts of a trial were asked to consent to further randomisation to receiving trial results by different methods.	Aggregate	Not specified
Cox 2011 [24]	Feedback of trial results to participants: a survey of clinicians’ and patients’ attitudes and experiences	Trial participants and HCP	81; 145	Pt 62.5% male; HCP 63.2 male	Pt mean 63.0 years HCP mean 47.8 years	Cancer networks	UK	The aim of this research was to explore the practice of feeding back trial results to those who take part in cancer trials.	Postal questionnaire survey	N/A	N/A
Dixon-Woods 2011 [25]	Providing the results of research to participants: a mixed-method study of the benefits and challenges of a consultative approach	Trial participants and stakeholders	16	Female	Not specified	Secondary care	UK	We aimed to develop, deliver, and evaluate a consultative approach to inform provision of feedback about research findings to participants in the Oracle Children Study (OCS).	An iterative process, including focus groups and consultation with OCS stakeholders to inform the development of a feedback package, including a results leaflet. A questionnaire survey of participants’ reactions to receiving the results leaflet was conducted.	Aggregate	Not specified
Locock 2011 [26]	Personal experiences of taking part in clinical trials—a qualitative study	Trial participants	42	27 female; 15 male	38-84 years	N/A	UK	To investigate people’s experiences of and attitudes to participation in clinical trials	interviews	N/A	N/A
Williams 2011 [27]	The Italian-American Clinical Trial of Nutritional supplements and Age-Related Cataract (CTNS)	Trial participants	610	46.7% female; 53.3% male	Mean age 80.5	Not specified	Italy	To determine whether participants were satisfied with the trial results.	Survey	Aggregate and individual	After publication
Darbyshire 2012 [28]	Disseminating results to clinical trial participants: a qualitative review of patient understanding in a post-trial population	Trial participants	40	Not specified	Not specified	Secondary care	UK	To identify the most appropriate format for results dissemination to maximise understanding of trial results.	Qualitative, postal questionnaire	Aggregate	Simultaneously with publication
Ferrierre 2012 [29]	Return of individual research results and incidental findings in the Clinical Trials Cooperative Group setting	Directors of Clinical Trials Cooperative Groups	10	Not specified	Not specified	N/A	USA	To establish some of the similarities and dissimilarities of general conduct of research and current opinions on treatment of IRRs and IFs in the Cooperative Group setting. (individual research results and incidental finding)	29 item survey	N/A	N/A
Getz 2012 [30]	Meeting the obligation to communicate clinical trial results to study volunteers	Trial participants And HCP	364; 10	Not specified	Not specified	Secondary care	USA	To assess the feasibility of a routine mechanism to communicate trial results and to test the process of setting volunteer expectations prior to disclosing the lay summaries.	Mixed methods—intervention, focus groups, phone interviews and a questionnaire Intervention—pre and posttest of understanding	Aggregate	Not specified
Mancini 2012 [31]	Transparency in the presentation of trial results may not increase patients’ trust in medical researchers	Trial participants	107	Female	Mean age 56.7 years	Secondary care	France	To investigate the effect on the participants’ TMRs of providing final trial results to participants via the Internet. (TRM – trust in medical researchers)	Mailed self-administered questionnaires	Aggregate	8 months after the first public disclosure of the conclusions
Sarradon-Eck 2012 [32]	“They should take time”: disclosure of clinical trial results as part of a social relationship	Trial participants	29	Female	Median 53 years	Secondary care	France	The aim of this qualitative study (Study 1) was to explain some of the findings obtained in the previous large psychosocial survey (Study 2) in which it was nested by examining them more closely from a different angle	Survey	Aggregate	Not specified
Armstrong 2013 [33]	Unblinding following trial participation: qualitative study of participants’ perspectives	Trial participants	38	Female	Mean age 39 years	Secondary care	UK	To explore trial participants’ perspectives on whether they would like to be unblinded as to the treatment arm to which they were allocated following involvement in a large randomised controlled trial (RCT).	Semi-structured interviews	Aggregate	Not specified
Chen 2015 [34]	Evaluating medical information’s potential advancement of clinical trial data sharing through lay summaries of results	HCP	31	Not specified	Not specified	N/A	Not specified	To assess appropriateness of, and make recommendations on Medical Information involvement in the creation and dissemination of lay summaries of clinical trial results.	Online survey	N/A	N/A
Tarrant 2015 [35]	Consent revisited: the impact of return of results on participant’ views and expectations about trial participation	Trial participants	38	Female	Mean age 39 years	Secondary care	UK	We explored participants’ views of their decision to consent to a clinical trial after they received results showing adverse outcomes in some arms of the trial.	Semi-structured interviews	Aggregate	Not specified
Elzinga 2016 [36]	Adult patient perspectives on clinical trial result reporting: a survey of cancer patients	Current or previous trial participants	189	37% male	Median age 60 years	Secondary care	Canada	To assess adult cancer patient preferences surrounding aggregate result disclosure to study participants.	46-item questionnaire	N/A	N/A
Dietrich 2017 [37]	Improving information exchange with clinical trial participants: a proposal for industry	Patients, and health care professionals	3045; 462	Not specified	Not specified	Not specified	Various	To capture the current status of information exchange and identify possible future practices between the major stakeholders within the clinical research ecosystem.	Patients, sponsors, sites, and HCPs were engaged through surveys, interviews, and/or advisory boards	N/A	N/A
Racine 2017 [38]	Participants’ perspectives and preferences on clinical trial results dissemination: the TRUST Thyroid Trial experience	Trial participants	123	48 female; 75 male	65–74 years 69; 75+ years 54	Secondary care	Ireland	The aim of this study is to use a patient and public involvement (PPI) approach to identify, develop and evaluate a patient-preferred method of receiving results of the Thyroid Hormone Replacement for Subclinical Hypo-Thyroidism Trial (TRUST).	Mixed methods study with three consecutive phases. Development Of a patient-preferred result method using semi-structured focus groups and a consensus-orientated-decision model, a PPI group to refine the method and adult literacy review for plain English assessment. Evaluated using an RCT.	Aggregate	Not specified
Scott 2018 [39]	Returning research results: caregivers’ reactions following computerised cognitive training among childhood cancer survivors	Caregivers	43	Not reported	Not reported	Secondary Care	USA	The current study explores the practice of returning research results to families of childhood cancer survivors that previously participated in the COGTRN study.	Survey	Aggregate	2 years after completion of study
Aldinger 2018 [40]	Returning aggregate results of clinical trials: empirical data of patient preferences	Trial participants and patients	211	survey 1 male 9 female 66 survey 2: male 48 female 77	survey 1: 18–30 *n*=1 31–50 *n*=15 51–70 *n*=51 >71 *n*=9 Survey 2: 18–30 *N*=16 31–50 *N*=32 51–70 *n*=65 >71 *n*=11	Secondary Care	USA	To investigate the expectations and preferences for sharing of aggregate clinical trial results.	Two surveys	Aggregate	
Lindquist 2019 [41]	Leveraging patient/community partnerships to disseminate patient centred outcomes research in geriatrics	Community partners and patients	10	All females	Ages ranging from 55 to 87 (with a mean age of 71.6 years, sd 8.2)	N/A	USA	To provide written guidance or ‘how to’ for future patient/community partners and researchers to disseminate patient-centred research.	Semi-structured interviews	N/A	N/A
Schroter 2019 [2]	Frequency and format of clinical trial results disseminated to participants: a survey of trialists	First authors of clinical trial papers	1818 (out of 3127 contacted)	Not specified	Not specified	N/A	71 different countries	To determine the frequency and format of dissemination to trial participants and wider patient communities, and to explore barriers to doing so.	Online survey	N/A	N/A

*N/A* Not applicable as included study did not disseminate results to participants as part of study design, *N/S* No information specified, *HCP* Health care professional

### Characteristics of included studies

**Fig. 2:**
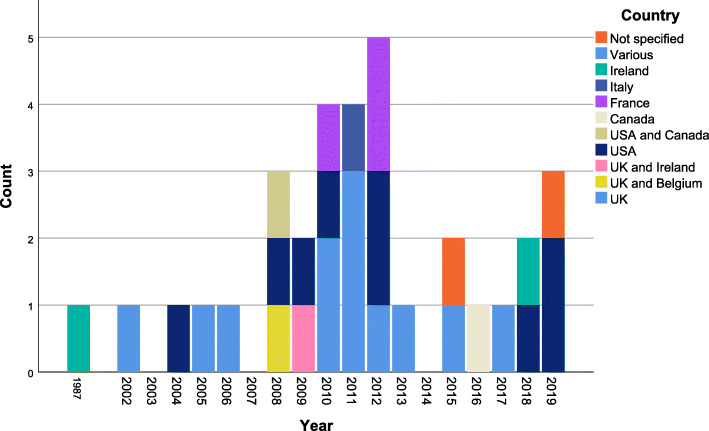
Geographical and temporal distribution of the included studies

The 33 studies we identified for inclusion were published between 1987 and 2019 with 70% (*n*=23) published between 2010 and 2019and five pre-2008 studies being identified from the previous review [4] (Fig. 2). Most studies (*n*=27, 82%) were conducted in single countries, namely the UK (*n*=11).

**Fig. 3:**
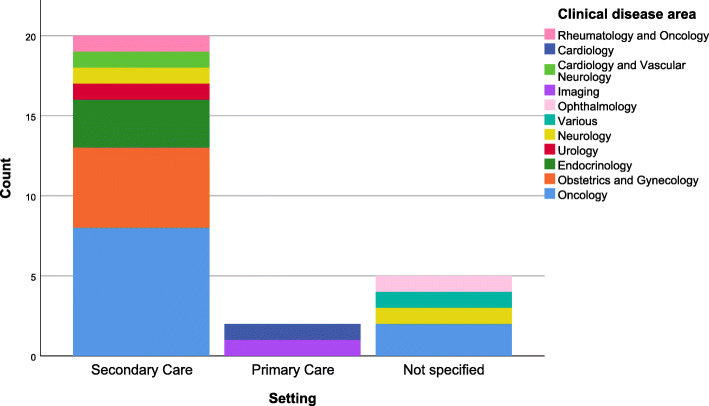
Clinical disease area and setting of included studies. A total of 6 studies are not included as they were not conducted within a clinical setting (*n*=3) or not within both a clinical disease and clinical setting (*n*=3)

The trial settings were situated across a broad range of clinical specialties and health areas but tended to focus more in secondary care settings and most frequently within oncology (*n*=11, 33%) (Fig. 3).

The median number of participants included in the eligible studies was 123 (range 10–3516) with a cumulative total of 12,700 participants across the 33 included studies. Of those that reported gender, ten included women only, two included men only, and eight included both men and women. Variability in reporting was also true for age which (where available) was most often reported as a median or mean, with the age of participants across reporting studies spanning from 18 to 80. Of the 33 included studies, nine (27%) reported on the ethnicity of study participants. The majority of participants in these studies were white, ranging from 83 to 98% (median 92%). Participants were from several trial stakeholder groups and included patients, trial participants (current or previous), health care professionals, sponsors, clinical trial unit directors, community partners, caregivers, and trial investigators. The review was very broad in its inclusion criteria with regard to study design and this is reflected in the types of studies included, some of which included mixed methods studies with various components. The methods each included study used to investigate aspects of dissemination of trial results varied, but of note only two were RCTs (Table 2).

**Table 2:** Main results or findings of included studies in terms of the ‘who, what and when’ of results dissemination

Study (first author and year of publication)	Type of results (aggregate/individual both)	Content of trial results communicated	Mode of delivery	Description of development of results information	Description of PPI and public involvement in development	Timing of feedback	Response rate/uptake of feedback
Garcia 1987 [10]	N/A	N/A	N/A	N/A	Not reported	N/A	N/A
Di Blasi 2002 [11]	N/A	Unblinding	N/A	N/A	Not reported	N/A	N/A
Partridge 2004 [12]	N/A	N/A	N/A	N/A	Not reported	N/A	N/A
Dinnett 2005 [13]	Individual	Unblinding	Telephone	The summary of the study results was prepared by a senior member of the PROSPER executive who had been unblinded and involved in the analysis of the primary data.	Not reported	Simultaneously with publication	1391/2520 (55%)
Dixon-Woods 2006 [14]	Aggregate	The leaflet explained that the results might remind participants of a difficult time, and offered the opportunity to contact the ORACLE team in case of any questions.	Postal	The results leaflet comprised a two page summary of the ORACLE findings, written in close collaboration with a consumer representative from the trial steering committee.	Written in close collaboration with a consumer representative from the trial steering committee.	Simultaneously with publication	All of the 8941 women who were recruited to ORACLE in the UK were offered the opportunity to request the trial results, 1803 (20% of all participants) requested this information.
Avins 2008 [15]	Aggregate	Using the tables and figures from the published manuscript	Face-to-face Publication	Not specified	Not reported	After publication	For the first meeting, 58 letters of invitation were mailed and 13 participants expressed interest in attending but only 3 participants actually attended the presentation (5%). For the second meeting, 167 invitations were mailed and 30 participants expressed a desire to attend though only 17 participants were present at the meeting (10%).
Dorsey 2008 [16]	Aggregate	A summary of the study’s results that included results of the primary and secondary measures and principal safety findings.	Media release Telephone Teleconference	Not specified	Not reported	Simultaneously with press release	114 out of 217—(52.5% )
Johnson 2008 [17]	N/A	N/A	N/A	N/A	Not reported	Not specified	N/A
Darbyshire 2009 [18]	Aggregate	N/A	Press release	Not specified	Not reported	Simultaneously with publication	N/A
Partridge 2009 [19]	Aggregate	The interim trial results and recommendation	Postal	Not specified	Not reported	Not specified	N/A
Brealey 2010 [20]	Aggregate	Two leaflets (short and long) summarising the trial findings, and a one-page questionnaire. The short leaflet was a one-page summary in the style of an abstract that was written in plain language using bullet points, with minimum use of numbers, and no pictures or diagrams. The longer leaflet was four pages in length which included a picture of MRI of the knee and two diagrams (a pie chart and bar chart) presenting results of the trial. The content of the leaflet was more technical with greater use of numbers and percentages and explained the main results in terms of confidence intervals.	Postal	Not specified	Not reported	After publication	Not specified
Dalal 2010 [21]	Aggregate	Summary of the study findings based on the published journal abstract, which was suitable for lay readers	Pt preference (Postal, electronic, face-to-face presentation, online)	A lay summary leaflet of the research results was prepared with the help of two patients with cardiac disease.	A lay summary leaflet of the research results was prepared with the help of two patients with cardiac disease.	After publication	Not specified
Getz 2010 [22]	Aggregate	Each of the formats presented the same core information identified by the CISCRP editorial translation team.	Face-to-face Online Audio	Translated the clinical trial results that were posted on http://clinicaltrials.gov into lay language	The CISCRP team included medical and consumer writers, graphic artists, web-page designers, as well as technical staff and professional voiceovers from Public Health Television.	After publication	Not specified
Mancini 2010 [23]	Aggregate	Not specified	Online	Not specified	Not reported	Not specified	Not specified
Cox 2011 [24]	N/A	N/A	N/A	N/A	Not reported	N/A	N/A
Dixon-Woods 2011 [25]	Aggregate	Reported the principal findings of the OCS, a reminder of the original ORACLE trial, the background, and reasons for the follow-up study, and the medical conditions and functioning problems that had been studied in the OCS. It further included basic explanations of clinical trials, placebos, and randomisation, as well as details of the scientific papers reporting the study findings.	Postal	Informed by the findings of the focus groups and input from other stakeholders, a results leaflet for participants was produced, and an integrated process of feedback and support was developed.	Not reported	Not specified	Not specified
Locock 2011 [26]	N/A	N/A	N/A	N/A	Not reported	N/A	N/A
Williams 2011 [27]	Aggregate and individual	The frequency figures were used to demonstrate the effect of treatment. The letter indicated that, because of the qualitatively different effect of treatment on the different types of cataract, the investigators could not recommend regular use of the supplement for the prevention of cataract. Efforts were made to present data in the letter so that they were understandable to a lay population. For example, instead of presenting hazard ratios and tests of significance as was done in the published paper, absolute frequencies of end-points in the study arms were presented.	Postal	The material for dissemination of the results to the participants (a letter and two questionnaires) was prepared by the Steering Committee.	The letter and enclosed questionnaire were shown to a small number of elderly patients to ensure that they were clear and comprehensible	After publication	The offer to reveal treatment assignment was accepted by 480 of 610 (78.7%) responders.
Darbyshire 2012 [28]	Aggregate	Headline results	Postal	N/A	Not reported	Simultaneously with publication	N/A
Ferrierre 2012 [29]	N/A	Not specified	N/A	N/A	Not reported	Not specified	Not specified
Getz 2012 [30]	Aggregate	Lay summary of clinical trial results	Postal Online Telephone	Lay summary based on technical summary developed for posting on ClinicalStudyResults.org and ClinicalTrials.gov.	Not reported	Not specified	Not specified
Mancini 2012 [31]	Aggregate	Not specified	Online	Not specified	Not reported	8 months after the first public disclosure of the conclusions	
Sarradon-Eck 2012 [32]	Aggregate	Not specified	Face-to-face	A medical journalist, with the help of expert patients from the French Cancer League, then wrote a patients’ leaflet explaining the trial results.	Not reported	Not specified	N/A
Armstrong 2013 [33]	Aggregate	The leaflet presented the results separately for the two conditions for which women were being treated in the trial—preterm rupture of the membranes (PROM) or spontaneous preterm labour (SPL). A covering letter reminded each woman which condition she presented with when she joined the ORACLE trial and directed her to the most relevant set of results within the leaflet.	N/A	Not specified	Not reported	Not specified	Not specified
Chen 2015 [34]	N/A	N/A	N/A	N/A	Not reported	N/A	N/A
Tarrant 2015 [35]	Aggregate	Headings in the leaflet -What the ORACLE Clinical Trial aimed to do -How the ORACLE Clinical Trial was designed -Groups in the ORACLE Clinical Trial -Results of the original ORACLE Trial: summary -Impact of the ORACLE Trial -The ORACLE Children Study -What was measured in the ORACLE Children Study -Results of the ORACLE Children Study -What is Cerebral Palsy? -Impact of the ORACLE Children Study -What is a clinical Trial? What is a placebo? Why is randomisation needed? Who can I contact? Where can I find the scientific papers?	Postal	Not specified	Not reported	Not specified	N/A
Elzinga 2016 [36]	N/A	N/A	N/A	Not specified	Not reported	N/A	N/A
Dietrich 2017 [37]	N/A	N/A	N/A	TransCelerate interviewed sponsors, conducted surveys with patients and HCPs, and conducted advisory boards with patients and sites to capture the current status and identify possible future practices related to information exchange	Not reported	N/A	N/A
Racine 2018	Aggregate	Headings: What was the TRUST Thyroid Trial? Who was in charge of the trial? What was the aim of the TRUST Trial? How long was the TRUST trial? Who took part in the TRUST trial? Why did you ask me to take part? What is subclinical hypothyroidism (SCH)? What are the symptoms of SCH? How is SCH diagnosed? How is SCH treated? What is Levothyroxine? What are the side effects of Levothyroxine? How was the trial carried out? What were the results of the TRUST trial? Should doctors treat people with subclinical hypothyroidism? What should I do now? Media release: TRIAL RESULTS European 5-year study of 737 older adults No worthwhile benefits from levothyroxine treatment About the TRUST research project	Postal	Iteratively developed a patient-preferred result method using semi-structured focus groups and a consensus-orientated-decision model, a PPI group to refine the method and adult literacy review for plain English assessment.	PPI group to refine the method and adult literacy review for plain English assessment.	Not specified	Feedback sent to all 101 participants
Scott 2018 [39]	Aggregate	Two-page summary consisted of 5 paragraphs, the first comprised a section thanking the families for their participation, disclosing publications and presentations that resulted from the study, and describing the benefits and risks of receiving the research results. Second, the study was summarised, including the hypotheses and methods. Results were then presented in a bulleted, easy to read format, then summarised in a brief paragraph, along with future directions. A section offering future neuropsychological assessment opportunities and contact information for researchers was provided. At the end of the summary, a reference to the survey was enclosed, along with publication citations.	Postal	The lay summary was developed based on the COG recommendations for returning a summary of research results	Not reported	2 years after completion of study	N/A
Aldinger 2018 [40]	Aggregate	Two summaries for separate trials were developed. Each plain-language summary began with a note of appreciation and a disclaimer that newer information may be available since the summary was completed. Study information in the template contained a summary of the study group and treatments, timeframe, and location of the sites. Next, the template described the study design and included a section on ‘side effects.’ The study results were then summarised with the disclaimer that they were limited to the particular study of the people who enrolled. Final comments included the official name of the study, the ClinicalTrials.gov unique identifier, the sponsor of the study, where further information could be found, and who to contact for additional information.	Online	Base on existing template that had been published [14] and its features incorporated into the European Union directive on plain-language summaries [15]. Each plain-language summary was prepared by a member of the study team and was reviewed and edited by another. Both of these individuals had been members or leaders of the multi-stakeholder group that developed the plain-language summary template. Each summary was then reviewed for accuracy and approved by the research teams of integrative medicine studies. Health literacy and numeracy principles were used throughout..” Each summary was then reviewed for accuracy and approved by the research teams of integrative medicine studies.	As a model for a plain-language summary of aggregate research results, we used a published template that had been developed and vetted by a multi-stakeholder group, including patient advocates.	N/A	N/A
Lindquist 2019 [41]	N/A	N/A	N/A	N/A	Not reported	N/A	N/A
Schroter 2019 [2]	N/A	N/A	N/A	N/A	Patient partners as study leads/team	N/A	N/A

*N/A* Not applicable as included study did not disseminate results to participants as part of study design, *N/S* no information specified

**Fig. 4:**
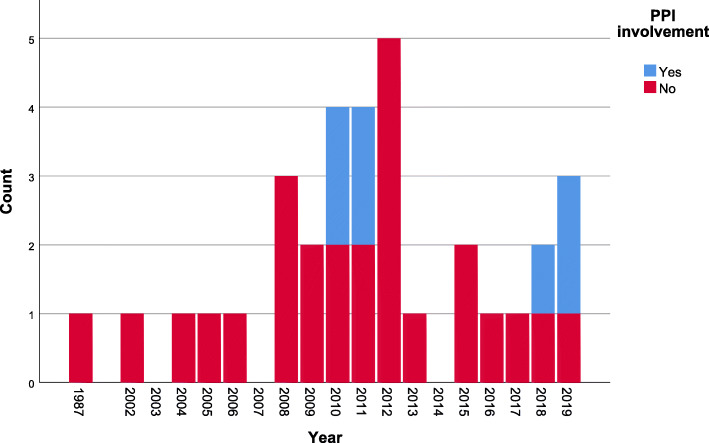
Temporal distribution of reported patient and public involvement in development of result materials and related procedures in included studies

Only four out of the 33 studies reported any patient or public involvement (PPI) in the development of results materials and related procedures (Fig. 4). Involvement tended to take the form of PPI partners contributing to the writing of, or approving, the summary leaflet of results (see Table 2).

### Key features of the trial result dissemination activity

**Fig. 5:**
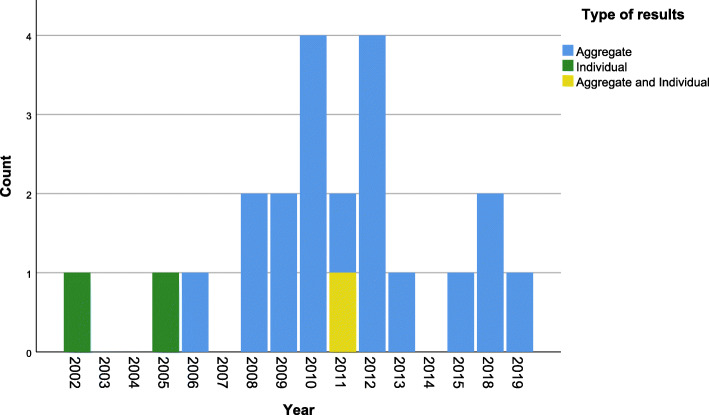
Temporal distribution of studies by level of trial results in dissemination in included studies. Eleven studies were not included as they do not report dissemination of results but rather explore attitudes towards, wishes for, or practice of dissemination of results. Aggregate, refers to whole trial level results. Individual, refers to individual participant level results

The majority of studies (*n*=19) were investigating the dissemination of aggregate (whole trial) level results with two studies considering provision of individual results and a further one considering both individual and aggregate (Fig. 5).

With regard to the content of the results shared with participants, some studies gave no or little detail, stating ‘lay summary of clinical trial results’ whereas others provided detailed section headings for the content (such as the aim of the trial, how it was designed, and key design features of placebo and randomisation) and some reports provided direct examples of the feedback provided. Several study reports indicated that the trial abstract or trial registry entry had been rewritten in ‘lay’ language but there were also examples of how published trial findings had been adapted for participants (e.g. absolute frequencies presented rather than hazard ratios). There was also variability across the included studies with regard to the description of how the information provided to trial participants was developed, with 11 studies reporting directly on the development of dissemination information (Table 2). For example, some studies reported working with a medical journalist to write the results leaflet, others reported adapting the technical summary into lay language (with and without patient input) and other studies conducted empirical research (using focus groups or surveys) to determine agreement on language used. Most included studies reported timing of provision of results as after peer-reviewed publication of trial results (*n*=7), or at publication (*n*=5), with other studies reporting after ‘first public disclosure’ (*n*=1), and several not specifying (Table 2). Only a handful of studies reported on what the response (i.e. if an evaluation questionnaire was issued about the results) or uptake to the offer of results (i.e. requested to have access to results) was, and the uptake or response ranged from 5 to 78% (Table 2).

**Fig. 6:**
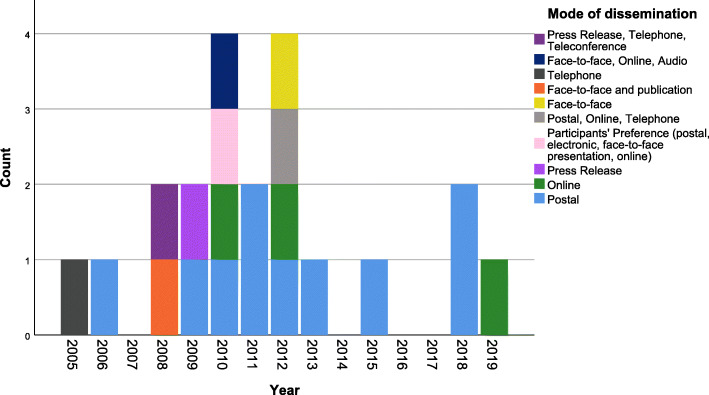
Temporal distribution of mode of dissemination of trial results in included studies. Twelve studies were not included as they did not report mode of delivery

Several modes of dissemination were reported across the included studies (Fig. 6). The most frequently used mode was postal (*n*=10), followed by online (*n*=3), face-to-face (*n*=1) and press release (*n*=1). Six studies reporting multiple methods (Fig. 6).

### Summary of main results or findings from included studies

Only seven studies explicitly reported on an intervention to improve dissemination being investigated or evaluated in their study. In two studies, participants were randomly allocated to an intervention (e.g. letter detailing the results or letter providing access to study results on website) versus a control (e.g. study results press release or no results letter). In the remaining five, participants received the trial results (through media release, phone call, thank you card, weblink) or were asked to assess a results ‘prototype before assessments of ‘effectiveness’ were made.

Outcomes reported included satisfaction with communication, preferences for information (content, presentation and length), understanding, whether participants discussed with others, trust in medical researchers, anxiety, guilt, anger, relief and whether results were helpful. The two RCTs comparing different methods for disseminating results measured different outcomes, trust or understanding. No studies explicitly assessed the acceptability to trial participants of receiving results from the trial they had participated in. However, there were reports of studies asking participants their preferences for receiving trial results (i.e. whether or not results were provided, or what mode of delivery).

Many positive features were reported by included studies with regard to dissemination of trial results to participants (see Table 3). Improvements in communication between health care teams and patients, and improvements in overall quality of care and satisfaction for patients were reported as benefits of dissemination of results to trial participants [2, 12, 22]. Demonstrating appreciation to participants for their contribution and increased accountability of researchers were cited as advantages [2, 12]. One study mentioned that dissemination of trial results may facilitate trial retention [26] and another cited raising public awareness of the importance of research [36]. The potential to motivate others to participate in future research was also identified as a benefit of disseminating trial results [2, 25, 27].

**Table 3:** Advantages and disadvantages of providing results

Study (author) and year	Interventions(s)	Description of outcomes (where appropriate)	Advantages	Disadvantages
Garcia, 1987 [10]	N/A	N/A	N/A	N/A
Di Blasi, 2002 [11]	N/A	N/A	Not Specified	Avoid biassing results at study follow up Avoid extra costs Avoid extra administrative work Difficulty contain people
Partridge, 2004 [12]	N/A	N/A	May ultimately improve communication between health care providers and patients, improve the quality of care delivered, and increase patient satisfaction with the care received as part of a clinical trial. Showing appreciation to patients. Courtesy to patients. Improving patient satisfaction with care or quality of life.	Not want to share unfavourable results with patients to avoid that treatment on a research study is designed to help future patients and not necessarily the individual patient on the study. Negative emotional effect on participants Participant difficulty understanding results Consumption of resources, including money and clinician time Respondents believed that an obligation to offer study results to patients would or might negatively effect their enrolment of patients on trials.
Dinnett, 2005 [13]			Not specified	Cost associated with unblinding—specifically staff salary time. Preparation, printing and distribution of letters, main study results and unblinding documents for primary care physicians and participants.
Dixon-Woods, 2006 [14]			Receiving a results leaflet through the mail satisfactory and preferable to personal contact to enable study at length and in private. Most of the comments on the content and format of the leaflet were positive. Half expressed feelings of pleasure on receiving the leaflet, particularly at what they saw as the success of the trial, or felt that taking part had been worthwhile.	One negative consequence of receiving the results was that for some women it revived memories of a difficult time.
Avins, 2008 [15]			Responses to provision of results largely positive and all respondents thought that future studies should include in person meetings.	Not specified
Dorsey, 2008 [16]	Media release from the investigators within a day after a sponsor-issued press release and a subsequent telephone call from the site staff to the participants; and conference call for research participants 2 weeks after the results were released.	Source and timing for learning study results and satisfaction with their communication.	Study participants reported high satisfaction with the telephone call and conference call but relatively low levels with the sponsor’s press release.	Not specified
Johnson, 2008 [17]	N/A	N/A	N/A	N/A
Darbyshire, 2009 [18]			Not specified	Concern that participants were unwilling to discuss their diabetes and treatment (allocated within a trial) with strangers from the same local area in an open meeting. Working age participants may require additional time off work to attend meeting or travel for elderly participants. Presenting results in English to individuals who do not have English as their first language.
Partidge, 2009 [19]			Most women felt they had been treated with dignity and respect during the trial. Women described their overall experience with the clinical trial up until the time of the survey as positive, and indicated that they would recommend participating in a clinical trial to someone else who had been diagnosed with cancer.	Anxiety should be considered, and psychosocial support may be required by some. Some women indicated that prior to receiving results they regretted participating in the clinical trial, and 4% of women regretted participation after receiving results.
Brealey, 2010 [20]	None	Preferences for information: content, presentation and length	Most participants preferred the four-page leaflet due to use of technical information and diagrams.	Not specified
Dalal, 2010 [21]			The majority of participants were happy with the method by which they received their results and the same proportion were pleased that they were informed.	A small proportion of patients indicated that they were upset by the results. One participant said: ‘My only criticism is that there seems to have been a long gap between completing the research and contacting me. I had thought that I had been forgotten.’
Getz, 2010 [22]			Study volunteers reacted positively to all three communication formats as did study staff. Study volunteers appreciated receiving information about their clinical trial and felt valued as contributors to the process of medical and scientific learning. Of the three formats, focus group participants considered the written report to be the most appealing. But noted the importance of receiving post-trial results communication in multiple formats to accommodate older people. Study volunteers want the clinical trial results summaries to be informative, easy to read, precise, not very wordy, with just the pertinent questions (who, what, when, where, why, and how) answered. Improvement in study volunteers’ confidence in their knowledge about the clinical trial, and understanding about their study’s objectives, side effects, and key findings. Study staff also reacted positively to being able to provide trial results summaries and in answering their questions.	Even after independently reviewing the trial summaries, focus group discussions revealed a number of areas where confusion remains among study volunteers
Mancini, 2010 [23]	Internet group received a letter stating that the trial results were available on the password-protected website, whilst the Control group received no letters	Participants expectations about the trial results, their preferences about the mode of disclosure, their declared uptake of the results and their understanding of the outcome of this trial. Whether patients discussed the results with their next of kin was also addressed.	Informing participants about the outcome of clinical trials would be useful and should be should be routine—either by patient’s request or physician’s discretion. Preferences about how patients should be informed about clinical trial results and by whom were expressed. Internet was less frequently preferred than a face-to-face consultation or a mailed letter. Oncologists, followed by trial investigators, were the participants’ preferred providers of trial result information. Participants reported discussing results with a close relative or others: such as spouse and other relatives, the oncologist, the general practitioner, and other patients. Talking about the results was said to be easier when they were perceived as positive. Negative results were also discussed, however, in order to obtain reassurance about their personal significance. The trial results were better understood by the Internet group than by the control group.	Not specified
Cox, 2011 [24]	N/A	N/A	Not specified	Negative psychological impact on patients/carers Negative results/bad news were the dominant concern
Dixon-Woods, 2011 [25]	None		Description is clear Results are interesting What did they feel when learned about results • Satisfaction • Concern • Both satisfaction and • concern • Indifference Appropriate to receive the results of the study by letter Recommend to other to take part	Participants found it particularly hard to understand the trial design and the methods of analysis. Many participants did not recall the aims or the findings of the original trial, and recommended that any feedback provided reminders of these. Use of language and numbers important to consider to avoid unanticipated effects of interpreting (and misinterpreting) the meaning of findings. Feedback development process was costly, staff salaries, consumables, the mail-out of the leaflets, recruiting and training staff for the helpline, etc.
Locock, 2011 [26]	N/A	N/A	May encourage retention Matter of interest, personal satisfaction, respect for contribution,	Not specified
Williams, 2011 [27]			Being informed of treatment assignment did not seem to discourage participants in the placebo arm, most of whom indicated that they would have certainly recommended participation in a similar trial to other people.	Delay between trial close out and study results being published. Ensuring trial participants were still alive and contact details were current were correct.
Darbyshire, 2012 [28]			Not specified	Not specified
Ferrierre, 2012 [29]	N/A	N/A	N/A	N/A
Getz, 2012 [30]	Thank you card and two reminder cards and trial results	Understanding of study (baseline) and comprehension of trial results	Overall, study volunteers agreed that it was ‘very’ or ‘somewhat’ important that they be thanked for their participation in a clinical trial; and indicated that they appreciated receiving a thank-you message following completion of the clinical trial.	Long delay between completing participation and receiving results
Mancini, 2012 [31]	Received letter informing them of the possibility of consulting a specific website to view the RCT final results.	Trust in medical researchers No difference between the groups		
Sarradon-Eck, 2012 [32]			Not Specified	Not Specified
Armstrong, 2013 [33]				Findings suggest a potential sense of frustration for participants at knowing the trial outcomes in aggregate, but not knowing their own treatment allocation Study also suggests caution to consider desire for unblinding is universal among trial participants. Some participants recognised that revelation of their treatment group could potentially bring risks as well as benefits, and that one of those risks was disruption of their existing narrative.
Chen, 2015 [34]	N/A	N/A	N/A	N/A
Tarrant, 2015 [35]			Women were able to reconcile their original decision to participate in the trial but there were several concerns.	Return of results led participants to question the basis of their decision to consent to the trial. Some were shocked at the outcomes of the research. They were distressed by the discovery that by taking part in the trial they had exposed their child to a possible risk of harm. This was associated with guilt, anger and a sense of betrayal by the maternity staff and researchers involved in the trial. Others experienced a profound breach of trust. They questioned the motives and actions of those involved in the research, feeling that they had been let down, misled or exploited when they were in a distressed and vulnerable position, by the very nursing and medical staff they trusted to care for them. They interpreted the negative outcomes as indicating that the doctors, nurses or researchers had not fulfilled their side of the co-operative bargain and experienced this as a sense of betrayal. One key implication of our study is the need for researchers to recognise that there will always be the potential for the return of results to cause distress or doubt.
Elzinga, 2016 [36]	N/A	N/A	May provide information to inform participant QoL Raise public awareness of importance of research Provide information to prevent future harm to participant Emphasise importance of participant to the research project Emphasise importance of participant to research in general Reduce secrecy surrounding research studies Decrease chance participant feels used by researcher	Distress caused by discovering participant was harmed by the research Distress for family/caregiver where the participant is deceased Distress caused by discovering participant was not helped by the research Distress caused by worries surrounding employability/insurability Distress caused by bringing up old memories and emotions Distress caused by guilt surrounding selection to better arm of the study
Dietrich, 2017 [37]	N/A	N/A	Not specified	A general lack of awareness of the true patient preferences in this area and their corresponding value was evident.
Racine, 2018	Patient preferred results letter or TRUST results press release	Patient understanding No difference between the groups	Participants wanted to receive results that are accessible and easy to understand. Preferred format is a letter.	No negative reactions
Scott, 2018 [39]	Participants’ caregivers were contacted and provided with a summary of results based on guidelines and survey.	Understanding Whether the summary was surprising or applicable to their child. Anxiety Satisfaction Guilt Anger Relief Whether they have others they can talk to about the results	Overall, most caregivers’ reactions to the summary were positive describing satisfaction with the results or importance of cognitive research. Results perceived as generally important, helpful and appropriately detailed. Several demonstrated an understanding of the summary New concerns or questions following summary included: lack of improvement in math skills and whether results would be long-term.	Caregivers explained feeling guilty ‘because [they would] like to do more for [their] child’ or ‘did not complete the last few sessions’ of the cognitive training.
Aldinger, 2018 [40]	Three-page, plain-language summary of results of study and a three half-page summary of the intervention study.	Whether summary was helpful	Reasons reported for sharing results: interest in research results—they wanted to learn what the study found; belief that it was an ethical expectation to share results with those who participated. Participants were interested if results had relevance to their own health.	Participants felt research teams should spend their time doing other activities rather than disseminating result. This is balanced/contingent on their contribution having helped someone else.
Lindquist, 2019 [41]	N/A	N/A	Important factors for dissemination: 1. Reaching out to those most affected by the results and their shared experiences with the results 2. Leveraging connections to the study population (e.g. parents, children, caregivers) 3. Determining the practical application of results Patients/community	Not specified
Schroter, 2019 [2]	N/A	N/A	Benefits of disseminating results to patients included supporting the spread of knowledge in the patient community, increased accountability for researchers, and an opportunity to empower patients. The potential to motivate people to participate in future research studies was noted by many and some suggested dissemination might encourage patients to consider interventions which could lead to better outcomes for them. It was also suggested that it might improve the doctor–patient relationship through building confidence and trust. Respondents further suggested that the impact of dissemination could be extended by giving patients the opportunity to share results within their own communities; there were mixed views on whether dissemination should be mandatory.	Some researchers said they do not think patients would be interested in receiving study results; others assumed not asking for them represented a lack of interest in getting them. Many were concerned that patients would lack the ability to understand the results and their implications.

*N/A* Not applicable as included study did not disseminate results to participants as part of study design

On the whole, when participants had received results this had been largely viewed as positive [14–16, 21, 25, 39]. Participants in some of the included studies identified preferences for receipt of results such as a preferred mode and length but recognised that a one-size-fits-all approach may not be appropriate (e.g. if population has a range of age groups) [16, 20, 22, 23, 25]. Some studies also reported improvements in understanding of trial results (based on pre and post test) once participants had been provided with the trial results [22, 39]. Participants also reported a feeling of ‘pleasure’ in what they viewed as contributing to a successful trial and that their contribution had been worthwhile and valued [2, 22, 25, 26, 36, 38]. This extended into one study that noted that participants felt it important for their contribution to be explicitly recognised through receipt of a thank you message [22]. Participants (in this case in a randomised evaluation of trial results in an ongoing trial) reported that results were easier to discuss if they were perceived as ‘positive’ and whilst ‘negative’ results were discussed this was to obtain reassurance [23]. Unblinding also did not seem to be a concern for some participants (in a trial of nutritional supplement compared to placebo), and they were not discouraged to continue if they were in the placebo arm [27].

Some studies identified a range of disadvantages, or cautionary considerations, for trial participants and/or health care professionals when disseminating results to trial participants (see Table 3). Health care professionals or researchers discussed concerns such as the potential extra costs both in terms of resource and time (theirs and others) to ensure the provision of results was done well [11, 13, 25]. They also expressed concerns about not wanting to share unfavourable results with participants as this would require further explanation about research being for the benefit of future patients rather than for those who participate [12]. Linked to this there were worries about the emotional effect of results, and participants’ understanding (both in terms of comprehension but also language barriers) [2, 12, 19, 28]. Health care professionals in one study stated that an obligation to provide results to participants would make them less likely to enrol patients [12]. Some researchers stated that participants did not want to receive results, and assumed if they did, they would ask for them [2].

Some of the disadvantages reported by trial participants were similar to health care professionals with impacts on their emotions either directly linked to trial results or linked to their initial recruitment into the trial due to a difficult time in their lives [14, 36]. When considering specific aspects of dissemination activity, some trial participants felt frustration at receiving aggregate results but not knowing individual results. In one case, some participants involved in a trial in pregnancy questioned their decision to consent to the trial and felt betrayed and angry towards trial and clinical staff when they had received results which they felt ill-informed to consider and which suggested mismatched expectations [33, 35]. Trial participants also raised frustrations at the delay between end of the trial and contact with the results. ‘Negative results’ or receiving bad news were cited as concerns by some [24]. Whilst not raised as a direct concern by participants, some of the included studies did identify areas where confusion remained amongst participants after receiving results [22, 25]. Logistical challenges linked to timelines of reporting but also ensuring participants were still alive and contact details were current were also cited as challenges [21, 22, 27].

## Discussion

This review is the first systematic mapping of the evidence to describe the characteristics of studies that have evaluated or explored dissemination of trial results to participants in phase III pragmatic effectiveness trials. We identified eligible studies published over the past 33 years, including a range of study designs, and set across a range of clinical areas and specialities. Overall, the scoping review has identified the largely ad hoc approach to research in this area, focussing on discrete activities required for particular trials; however, more recent studies are taking a broader approach to assessing the problem and considering solutions such as the recent survey by Schroter et al. [2].

There are several gaps highlighted by the evidence map both in terms of areas for replication and those for initiation of new research. Firstly, with regard to replication, we identified only two trials (or SWATs) of results dissemination methods, both assessing different interventions and both measuring different outcomes. A programmatic approach to replicating interventions (that assess aspects of content and mode of delivery and are specified using TidIER) and measuring the same outcomes (identified as important to a range of stakeholders) is required if these types of studies are to contribute meaningfully to the evidence base [42]. With regard to new research, identifying what aspects of information should generally be considered core for inclusion in trial result summaries for participants would be helpful. Similar studies identifying what information potential trial participants and research nurses consider core for participant information leaflets have provided helpful summaries for consideration when designing patient-facing trial information; this could be extended to trial results [43]. In addition, better understanding of the barriers and enablers for trial teams in their ability and intention to disseminate results could help to provide solutions for how to improve current practice. Emerging studies are demonstrating that whilst UK trial teams intend to disseminate results at start up, this activity often is not implemented or at least not in a way that is appropriate for the participants to engage with [2, 3]. Attention also needs to be given to determining what constitutes an appropriate approach to dissemination as this may vary across trials involving different populations, assessing different kinds of intervention for different kinds of health problem, and perhaps with different types of findings.

When considering who the results are for, the lack of ethnic diversity from those studies that reported their sample characteristics should be noted. Also, the lack of reporting of other characteristics, such as religion or sexuality, for which the reporting of results might need to consider and be sensitive to. Irrespective, ensuring trialists communicate results in culturally sensitive ways is also a key consideration going forwards. Another important area for investigation is how dissemination of results may need to be different in low- and middle-income countries, none of which were identified in our review, and adapted to a range of cultural contexts. There are some examples of this happening in African countries, with dissemination of findings through community events with song and dance [44, 45]. This has particular salience given a 2018 research prioritisation exercise on methodological research for global health trials identified ‘Methods of dissemination of findings’ as number 7 (out of 27) and far above recruitment and retention (at 18 and 17, respectively) [46].

The results of this review also identify that only a minority of studies conducted in this area explicitly stated that they actively included patients or the public as partners in the development of the dissemination of results. Previous research has shown that amongst trial teams who intend to disseminate results to trial participants, most (60%) intend to involve patients and the public in the dissemination activity [47]. It is likely that this involvement happens in practice much more than is reported in the literature on results dissemination. However, a failure to share that knowledge on what worked well and what could be improved contributes to research waste.

Whilst the majority of studies reported that study participants viewed disseminating results as a positive endeavour that should be encouraged, the notion that it can be done poorly, and have poor consequences, including some that should be recognised as harms was also identified across several studies. Concerns were raised, for example about the difficult experiences of finding out that a trial had ‘negative’ findings, or that as a participant they had received an inferior treatment, or that providing results reminded them of a difficult time in their lives, or further, that they regretted their decision to participate in the trial in the first place. Ensuring trial teams have safeguards in place to protect against these unintended consequences of disseminating results is critical to ensure that the action has the intended effect. Considering early on with patient/public partners what to share, when and how, could help to address some of these concerns.

### Strengths and limitations

This scoping review benefited from systematic methods which were guided by the general principles of the Cochrane handbook for systematic reviews intended to increase rigour and reduce bias [48]. As such, one of the key strengths of this review is the methods employed through each stage of study identification and extraction. In addition, the review identified a range of study designs and perspectives on dissemination of results and allows some level of aggregative summary to be synthesised. We also consulted with a range of relevant stakeholders (patients, methodologists, bioethicists, policy makers) via our advisory group who were key in helping ensure the scope of the review was relevant for current practice.

One of the limitations of the search was that we were limited to English language studies only and the search could have been enhanced through contacting known authors, etc. Therefore, there could be studies published in languages other than English that we have missed. Other limitations of our review are linked directly to the lack of complete reporting by the included studies. For example, it was not clear in all studies to explore perceptions of receiving results whether participants who were involved were only those who had opted to receive them in the first place. It would also be important in future research to explore the views of those who opted not to receive trial results. Finally, the review is likely subject to reporting bias in that many trials will report results back to participants and may have investigated that process but have not published and as such cannot be included in the review.

## Conclusions

This scoping review has identified several studies that have explored the ‘what’ and ‘how’ of disseminating results of trials to those who participated. However, few high-quality evaluative studies have been conducted that can provide evidence on the best ways to deliver this activity. Indeed, the literature also shows heterogeneity around what outcomes are measured and reported and further still, are important to, relevant stakeholders. Identifying these and conducting further replications of existing evaluations would add significantly to the evidence base. Whilst there are some very in depth qualitative studies focussing on dissemination of results, these have tended to be conducted in a few discrete clinical areas and as such, opportunities to extend this work into other areas and to consider how findings and recommendations do and do not generalise across different trial contexts should also be considered. The learning from these studies can be used as a platform for further research and to consider some core guiding principles of the opportunities and challenges when disseminating trial results to those who participated.

### Supplementary Information

**Additional file 1.**

## Data Availability

The datasets generated and/or analysed during the current study are available from the included studies details of which are provided in the reference section. No additional data was requested from authors.

## Appendix

### Search strategy

#### Embase; Ovid MEDLINE(R); PsycINFO

1. exp Clinical Trials as Topic/ use ppez (335805)
2. exp “clinical trial (topic)”/ use emef (259703)
3. Clinical Trials/ use psyc4, psyc5, psyc6, psyc7, psyc8, psyc9, psyc10, psyc11, psy12, psyc13 (9596)
4. (trial$ adj5 results).tw. (167299)
5. (research adj5 results).tw. (91272)
6. results.kw. (7042)
7. or/1-6 (817067)
8. Information dissemination/ (34210)
9. disclosure/ use ppez (13102)
10. interpersonal communication/ use emef (120985)
11. “DEBRIEFING (EXPERIMENTAL)”/ use psyc4, psyc5, psyc6, psyc7, psyc8, psyc9, psyc10, psyc11, psy12, psyc13 (67)
12. (results adj3 (disseminat$ or disclos$ or communicat$ or inform$ or provid$ or feedback or return$ or shar$ or receiv$ or send$)).tw. (386171)
13. or/8-12 (546925)
14. exp research subjects/ use ppez (17124)
15. Research Subject/ use emef (5607)
16. Experimental Subjects/ use psyc4, psyc5, psyc6, psyc7, psyc8, psyc9, psyc10, psyc11, psy12, psyc13 (1734)
17. (participants or subjects).kw. (2707)
18. ((participant$ or subject$ or patient$ or volunteer$) adj5 results).tw. (2081910)
19. or/14-18 (2105949)
20. 7 and 13 and 19 (3330)
21. 20 not (letter or comment or note).pt. (3226)
22. limit 21 to yr=“2008 –Current” (2695) (MEDLINE 663, EMBASE 1725 PSYCINFO 307)

#### CINAHL (www.ebscohost.com/)

- S4 S1 AND S2 AND S3 Limiters - Published Date: 20080101-20171231 (*N*=244)
- S3 (MH “Research Subjects”) OR TX ( results N5 (participant* or subject* or patient* or volunteer*) )
- S2 TX ( results N3 (disseminat* OR disclos* or communicat*) ) OR TX ( results N3 (communicat* or inform* or provid* or feedback) ) OR TX ( results N3 (return* or shar* or receiv* or send*) )
- S1 TX trial* N5 results OR TX research N5 results

## References

[1] https://www.wma.net/policies-post/wma-declaration-of-helsinki-ethical-principles-for-medical-research-involving-human-subjects/. 2013. Accessed 25 Nov 2020.

[2] Schroter S, Price A, Malički M, Richards T, Clarke M (2019). Frequency and format of clinical trial results dissemination to patients: a survey of authors of trials indexed in PubMed. BMJ Open.

[3] Raza MZ, Bruhn H, Gillies K (2020). Dissemination of trial results to participants in phase III pragmatic clinical trials: an audit of trial investigators intentions. BMJ Open.

[4] Shalowitz DI, Miller FG (2008). Communicating the results of clinical research to participants: attitudes, practices, and future directions. PLoS Med.

[5] Long CR, Purvis RS, Flood-Grady E, Kimminau KS, Rhyne RL, Burge MR, Stewart MK, Jenkins AJ, James LP, McElfish PA (2019). Health researchers’ experiences, perceptions and barriers related to sharing study results with participants. Health Res Policy Syst.

[6] https://www.hra.nhs.uk/planning-and-improving-research/policies-standards-legislation/research-transparency/. Accessed 25 Nov 2020.

[7] Grimshaw J (2020). A guide to knowledge synthesis: a knowledge synthesis chapter.

[8] Peters MDJ, Marnie C, Tricco AC, Pollock D, Munn Z, Alexander L, McInerney P, Godfrey CM, Khalil H (2021). Updated methodological guidance for the conduct of scoping reviews. JBI Evid Implement.

[9] https://www.researchregistry.com/browse-the-registry#home/registrationdetails/5af98c264051862a535ce5ec/. Accessed 25 Nov 2020

[10] Garcia J (1987). Sharing research results with patients: the views of care-givers involved in a randomized controlled trial. J Reprod Infant Psychol.

[11] Di Blasi Z, Kaptchuk TJ, Weinman J, Kleijnen J (2002). Informing participants of allocation to placebo at trial closure: postal survey. BMJ.

[12] Partridge AH, Hackett N, Blood E, Gelman R, Joffe S, Bauer-Wu S, Knudsen K, Emmons K, Collyar D, Schilsky RL, Winer EP (2004). Oncology physician and nurse practices and attitudes regarding offering clinical trial results to study participants. J Natl Cancer Inst.

[13] Dinnett EM, Mungall MM, Kent JA, Ronald ES, McIntyre KE, Anderson E, Gaw A, PROSPER Study Group (2005). Unblinding of trial participants to their treatment allocation: lessons from the Prospective Study of Pravastatin in the Elderly at Risk (PROSPER). Clin Trials.

[14] Dixon-Woods M, Jackson C, Windridge KC, Kenyon S (2006). Receiving a summary of the results of a trial: qualitative study of participants’ views. BMJ.

[15] Avins AL, Bent S, Padula A, Staccone S, Badua E, Goldberg H (2008). Initial experience with a group presentation of study results to research participants. Trials.

[16] Dorsey ER, Beck CA, Adams M, Chadwick G, de Blieck EA, McCallum C, Briner L, Deuel L, Clarke A, Stewart R, Shoulson I, Huntington Study Group TREND-HD Investigators (2008). Communicating clinical trial results to research participants. Arch Neurol.

[17] Johnson L, Barrett-Lee P, Ellis P, Bliss JM, TACT Trial Management Group (2008). How do patients want to learn of results of clinical trials? A survey of 1431 breast cancer patients. Br J Cancer.

[18] Darbyshire JL, Holman RR, Price HC (2009). Presenting the results of clinical trials to participants. Clin Med (Lond).

[19] Partridge AH, Wolff AC, Marcom PK, Kaufman PA, Zhang L, Gelman R, Moore C, Lake D, Fleming GF, Rugo HS, Atkins J, Sampson E, Collyar D, Winer EP (2009). The impact of sharing results of a randomized breast cancer clinical trial with study participants. Breast Cancer Res Treat.

[20] Brealey S, Andronis L, Dennis L, Atwell C, Bryan S, Coulton S, Cox H, Cross B, Fylan F, Garratt A, Gilbert F, Gillan M, Hendry M, Hood K, Houston H, King D, Morton V, Robling M, Russell I, Wilkinson C (2010). Participants’ preference for type of leaflet used to feed back the results of a randomised trial: a survey. Trials.

[21] Dalal H, Wingham J, Pritchard C, Northey S, Evans P, Taylor RS, Campbell J (2010). Communicating the results of research: how do participants of a cardiac rehabilitation RCT prefer to be informed?. Health Expect.

[22] Getz K, Oullette E, Simmons D, Morrison BW, Scott N, Wilenzick M, Jones N (2010). Providing results to volunteers. Appl Clin Trials.

[23] Mancini J, Genre D, Dalenc F, Ferrero JM, Kerbrat P, Martin AL, Roché H, Maylevin F, Tarpin C, Viens P, Genève J, Julian-Reynier C (2010). Participants’ uptake of clinical trial results: a randomised experiment. Br J Cancer.

[24] Cox K, Moghaddam N, Bird L, Elkan R (2011). Feedback of trial results to participants: a survey of clinicians’ and patients’ attitudes and experiences. Eur J Oncol Nurs.

[25] Dixon-Woods M, Tarrant C, Jackson CJ, Jones DR, Kenyon S (2011). Providing the results of research to participants: a mixed-method study of the benefits and challenges of a consultative approach. Clin Trials.

[26] Locock L, Smith L (2011). Personal experiences of taking part in clinical trials - a qualitative study. Patient Educ Couns.

[27] Williams SL, Ferrigno L, Maraini G, Rosmini F, Sperduto RD (2011). A post-trial survey to assess the impact of dissemination of results and unmasking on participants in a 13-year randomised controlled trial on age-related cataract. Trials.

[28] Darbyshire JL, Price HC (2012). Disseminating results to clinical trial participants: a qualitative review of patient understanding in a post-trial population. BMJ Open.

[29] Ferriere M, Van Ness B (2012). Return of individual research results and incidental findings in the clinical trials cooperative group setting. Genet Med.

[30] Getz K, Hallinan Z, Simmons D, Brickman MJ, Jumadilova Z, Pauer L, Wilenzick M, Morrison B (2012). Meeting the obligation to communicate clinical trial results to study volunteers. Expert Rev Clin Pharmacol.

[31] Mancini J, Genre D, Dalenc F, Maylevin F, Martin AL, Viens P, Julian-Reynier C (2012). Transparency in the presentation of trial results may not increase patients’ trust in medical researchers. Clin Trials.

[32] Sarradon-Eck A, Sakoyan J, Desclaux A, Mancini J, Genre D, Julian-Reynier C (2012). “They should take time”: disclosure of clinical trial results as part of a social relationship. Soc Sci Med.

[33] Armstrong N, Jackson CJ, McNicol S, Dixon-Woods M, Kenyon S, Tarrant C (2013). Unblinding following trial participation: qualitative study of participants’ perspectives. Clin Trials.

[34] Chen M, Lentz C, Chang C, Whiteside K, Faria T, Berg T (2015). Evaluating medical information’s potential advancement of clinical trial data sharing through lay summaries of results. Poster presented at the 26th Annual DIA Medical and Scientific Communications Forum in Glendale, AZ.

[35] Tarrant C, Jackson C, Dixon-Woods M, McNicol S, Kenyon S, Armstrong N (2015). Consent revisited: the impact of return of results on participants’ views and expectations about trial participation. Health Expect.

[36] Elzinga KE, Khan OF, Tang AR, Fernandez CV, Elzinga CL, Heng DY, Vickers MM, Truong TH, Tang PA (2016). Adult patient perspectives on clinical trial result reporting: a survey of cancer patients. Clin Trials.

[37] Dietrich J, Alivojvodic J, Seliverstov I, Metcalf M, Jakee K (2017). Improving information exchange with clinical trial participants: a proposal for industry. Ther Innov Regul Sci.

[38] Racine E, Hurley C, Cheung A, Sinnott C, Matvienko-Sikar K, Smithson WH, Kearney PM (2017). Study within a trial (SWAT) protocol. Participants’ perspectives and preferences on clinical trial result dissemination: the TRUST Thyroid Trial experience. Contemp Clin Trials Commun.

[39] Scott SM, Ashford JM, Clark KN, Martin-Elbahesh K, Conklin HM (2018). Returning research results: caregivers’ reactions following computerized cognitive training among childhood cancer survivors. Neurooncol Pract.

[40] Aldinger CE, Ligibel J, Shin IH, Denninger JW, Bierer BE (2018). Returning aggregate results of clinical trials: empirical data of patient preferences. J Clin Transl Sci.

[41] Lindquist LA, Seltzer A, Forcucci C, Wong N, Ramirez-Zohfeld V (2019). Leveraging patient/community partnerships to disseminate patient centered outcomes research in geriatrics. Geriatrics (Basel).

[42] Hoffmann TC, Glasziou PP, Boutron I, Milne R, Perera R, Moher D, Altman DG, Barbour V, Macdonald H, Johnston M, Lamb SE, Dixon-Woods M, McCulloch P, Wyatt JC, Chan AW, Michie S (2014). Better reporting of interventions: template for intervention description and replication (TIDieR) checklist and guide. BMJ.

[43] Innes K, Cotton S, Campbell MK, Elliott J, Gillies K (2018). Relative importance of informational items in participant information leaflets for trials: a Q-methodology approach. BMJ Open.

[44] Supercharger M (2015). Now I know.

[45] https://www.fhi360.org/sites/default/files/media/documents/Communications%20Handbook%20for%20Clinical%20Trials.pdf. Accessed 25 Nov 2020.

[46] Rosala-Hallas A, Bhangu A, Blazeby J, Bowman L, Clarke M, Lang T, Nasser M, Siegfried N, Soares-Weiser K, Sydes MR, Wang D, Zhang J, Williamson PR (2018). Global health trials methodological research agenda: results from a priority setting exercise. Trials.

[47] Staley K, Elliott J (2017). Public involvement could usefully inform ethical review, but rarely does: what are the implications?. Res Involv Engagem.

[48] Higgins JPT, Thomas J, Chandler J, Cumpston M, Li T, Page MJ, Welch VA, editors. Cochrane handbook for systematic reviews of interventions,. 2nd Edition. Chichester: Wiley; 2019.

